# Intraoperative pneumatic tourniquet application reduces soft-tissue microcirculation, but without affecting wound healing in calcaneal fractures

**DOI:** 10.1186/s40001-024-01996-0

**Published:** 2024-09-17

**Authors:** Philipp Lichte, Felix M. Bläsius, Bergita Ganse, Boyko Gueorguiev, Torsten Pastor, Sven Nebelung, Filippo Migliorini, Kajetan Klos, Ali Modabber, Mario F. Scaglioni, Clemens Schopper, Frank Hildebrand, Matthias Knobe

**Affiliations:** 1 Department of Orthopaedics, Trauma and Reconstructive Surgery, University Hospital RWTH Aachen, Aachen, Germany; 2 Werner Siemens Foundation Endowed Chair of Innovative Implant Development, Saarland University, Homburg, Germany; 3 Department of Trauma, Hand and Reconstructive Surgery, Saarland University, Homburg, Germany; 4 AO Research Institute Davos, 7270 Davos, Switzerland; 5 Department of Orthopaedic and Trauma Surgery, Cantonal Hospital Lucerne, 6000 Lucerne, Switzerland; 6 Department of Diagnostic and Interventional Radiology, Aachen University Hospital, 52074 Aachen, Germany; 7 Gelenkzentrum Rhein-Main, Hochheim, Germany; 8 Department of Trauma, Hand and Reconstructive Surgery, Jena University Hospital, Friedrich Schiller University Jena, 07747 Jena, Germany; 9 Department of Oral and Maxillofacial Surgery, University Hospital of Aachen, Aachen, Germany; 10 Department of Hand- and Plastic Surgery, Lucerne Cantonal Hospital, Lucerne, Switzerland; 11 Department for Orthopedics and Traumatology, Kepler University Hospital GmbH, Johannes Kepler University Linz, Linz, Austria; 12 Medical Faculty, University of Zurich, 8091 Zurich, Switzerland; 13 Medical Faculty, RWTH Aachen University Hospital, 52074 Aachen, Germany; 14 Department of Orthopaedic Trauma, Westmuensterland Hospital, 48683 Ahaus, Germany

**Keywords:** O2C, Trauma, Blood supply, Injury, Capillaries, Tissue hypoxia, Foot surgery, Spectrophotometry

## Abstract

**Background:**

Wound healing complications are a major challenge following the extended lateral approach in calcaneal fractures. Soft-tissue microcirculation plays an important role via the delivery of oxygen, nutrients, and the regulation of a local milieu. *The aim of this clinical study was to examine the effect of intraoperative pneumatic tourniquet application on skin and subcutaneous microcirculation, and its impact on wound healing progression.*

**Methods:**

Patients *with calcaneal fractures were randomly assigned to two groups defined by a surgery conducted either with use or without use of a tourniquet. Blood flow (BF [AU]), tissue oxygen saturation (SO*_*2*_*[%]) and the relative amount of haemoglobin (rHb[AU]) were intraoperatively measured at two depths (2 and 8 mm) non-invasively by spectrophotometry (Micro-Lightguide O2C*^*®*^*, LEA Medizintechnik, Giessen, Germany).* Time points were before and after inflation of the pneumatic tourniquet and also at the end of surgery before deflation. A linear mixed model (LMM) was fitted for statistical analysis.

**Results:**

Thirty-four patients (3 women and 31 men) with 37 calcaneal fractures were included. In 22 of them, the surgery was conducted with a tourniquet and in the other 15 without its use. A significant decrease of microcirculation, characterized by decreases in blood flow (p = 0.011) and tissue oxygenation (p = 0.023) was measured in 8 mm depth after inflating the tourniquet. However, these changes did not influence the time of postoperative wound healing.

**Conclusion:**

The use of a pneumatic tourniquet reduces deep microcirculation without affecting postoperative wound healing.

*Trial registration* The study was registered in www.ClinicalTrials.gov (NCT01264146).

## Background

Calcaneal fractures *account for* 1–2% of all fractures and represent over 60% of all fractures occurring in the tarsal bones [1–3]. As a consequence of a high-energy injury mechanism and the thin soft-tissue cover, swelling and consecutive soft-tissue complications are frequent in the early period. In addition, chronic pain and long-term disability due to posttraumatic arthritis are common long-term health economic concerns [4].

Current treatment schemes include different operative treatment strategies as well as nonoperative management depending on the patient and the type of injury [4, 5]. On the one hand after open reduction and external fixation (ORIF) using the extended lateral approach higher complication rates are evident versus nonoperative treatment (23% versus 4%, respectively). On the other hand, higher functional outcome scores and lower rates of posttraumatic subtalar arthritis may favour operative treatment [1, 4–6]. To preserve the vulnerable soft-tissue cover over the lateral side of the hind foot and to avoid wound healing disorders, several minimally invasive techniques have been developed [7]. However, despite these innovative new surgical techniques, the best treatment strategy for displaced calcaneal fractures (Sanders type ≥ 2) remains controversial [4, 5, 8, 9] and ORIF through an extended lateral approach is still widely used [7, 10, 11].

Several risk factors *related to* the occurrence of wound healing problems—such as diabetes mellitus, smoking, open fracture, wound closure technique, fracture severity and an impaired microcirculation—have been identified [10, 12–15]. Sufficient tissue perfusion that provides oxygen to the injured tissue is essential for wound healing. In this context, preoperative tissue oxygen saturation was reported to be an independent predictive factor for wound healing complications [16]. On molecular level, the relationship between alterations of the microcirculation and the infection risk are well studied [17], whereas clinical evidence for this relationship is still missing for most body regions and injury types. Microcirculation is affected by multiple factors including trauma and fracture reduction for definitive care. Trauma regularly leads to decreases in oxygen supply with hypoxia, acidosis, as well as to local accumulation of metabolites with consecutive oxidative stress [18–20]. The ability for substrate delivery and metabolite removal is a well-known factor for determination of the local capillary supply [21]. In addition, the impairment of local immune functions supports bacterial growth [18].

Following an expectation for potential harmful influence, pneumatic tourniquets are used frequently to reduce blood loss during surgery and to ensure optimal conditions for fracture reduction. However, the effect of tourniquet application on tissue microcirculation during ORIF of calcaneal fractures and potential risks of the application of these devices are still unknown to date.

The aim of this clinical study was to examine whether (1) intraoperative use of a pneumatic tourniquet causes changes in blood flow, tissue oxygen saturation and haemoglobin content, and (2) whether these intraoperative observations affect wound healing during the postoperative clinical course.

## Methods

This prospective cohort study was approved prior to initiation by the local institutional review board (approval number EK 346/14). It was conducted according to the Declaration of Helsinki, including oral and written informed consent by all patients and registered at www.ClinicalTrials.gov (NCT01264146).

### Patients

All patients with calcaneal fractures who were treated at our academic level 1 trauma centre in a 3-year timespan were prospectively included in the study. Inclusion criteria were isolated calcaneal fractures and patient's age of at least 18 years. Exclusion criteria considered existence of open fractures, previous surgical treatment of the hindfoot in the patient’s history, peripheral arterial disease [chronic limb-threatening ischaemia (CLTI)], insulin-dependent diabetes mellitus, and hypercapnia. Baseline patient characteristics were collected at the admission. The clinical course included initial assessment in the emergency department, hospitalization to ensure tissue monitoring, and surgical treatment. After surgery the patients remained splinted and stayed on the orthopaedic and traumatology wards. The injured leg was positioned on an elevated pillow. Timing of surgery depended on the level of swelling evaluated by the wrinkle sign.

### Measurements

In all patients, a standardized laser-Doppler examination (O2C, LEA-Medizintechnik GmbH, Giessen, Germany) was performed along the incision of the extended lateral approach to the calcaneus at five locations, 2.5 cm apart from each other by the same examiner (MK) as *previously* described [16]. Time points are presented in Table 1. The probe was held in each location for 10s. A mean value was computed by the device and used for statistical analyses. The measurement principles and technical details of the O2C device were described previously [16, 22, 23]. The spectrometry delivers the capillary venous oxygen saturation (SO2; %) and the relative haemoglobin concentration [rHb; AU (arbitrary units)], and the laser-Doppler velocimetry quantifies the relative blood flow (BF; AU) in skin depths of 2 mm (superficial layer) and 8mm (deep layer).

**Table 1:** Time points of measurements

	Pneumatic tourniquet	No tourniquet
MTP 1	Before pneumatic tourniquet inflation	Before incision
MTP 2	Directly after incision (pneumatic tourniquet inflated)	Directly after incision
MTP 3	Directly before deflation and wound closure	Before wound closure

### Surgical intervention

All surgeries were performed by the senior author (MK). The use of the inflatable pneumatic tourniquet was randomly assigned directly before surgery. The tourniquet was inflated with 350 mmHg pressure before skin incision. A typical extended lateral approach was applied as previously described [16]. The preparation was performed directly on the bone, forming a skin flap mobilized distally and dorsally to expose the calcaneus. After preparation of the subtalar joint, the fracture was reduced and internal fixation was performed with a titanium calcaneal plate with 3.5/4.0 variable-angle locking screws (Königsee Implantate GmbH, Allendorf, Germany). In the tourniquet group, the tourniquet was deflated immediately after the osteosynthesis and haemostasis were performed before rinsing of the surgical site with sterile 0.9% saline solution. Subsequently, the wounds were closed via single interrupted stitch suture.

### Postoperative treatment

Postoperative immobilization was provided by a split plaster cast. Mobilization with partial weight-bearing of 10 kg was started at the first day after surgery and continued for 10 weeks. 5000 IU dalteparin sodium were injected daily to prevent thrombosis beginning six hours after completion of surgery until full weight-bearing. The wound was examined daily, beginning on the 2nd postsurgical day, and in cases of normal healing, the sutures were removed after 14 days. In case of persistent secretion, the sutures were removed another 7 days later. Outpatient clinic visits were scheduled weekly until week 12 and then monthly until 6 months after surgery.

### Study endpoint

*The primary endpoint of our study was the assessment of the intraoperative microcirculation. The secondary endpoint was prolonged wound healing which* was defined as persistent drainage later than on the seventh postoperative day and/or *presence of* necrotic wound edges. The diagnosis was *set* by a specialist in orthopaedic trauma surgery.

### Statistical analysis

Statistical analysis was performed using SPSS software package (v27.0, IBM, Armonk, NY, USA). A linear mixed model (LMM) was fitted to analyse paired repeated measurements of BF, SO_2_ and rHb at each depth (2 and 8 mm). Usage of a pneumatic tourniquet (yes/no) and measurement time points (MTP) two and three (see Table 1) were defined as fixed effects. MTP 1 right before the start of the surgery was defined as a random effect (baseline). An autoregressive covariance structure AR (1) was applied. Missing values were taken into account by a likelihood-based approach within the framework of mixed linear models under the assumption that missing values occur at random. Univariable logistic regression was used to evaluate the predictive value of BF, SO_2_ and rHb values for prediction of wound healing disorders (yes/no). The results should be interpreted against the presumption of no correction for possible confounders due to the small sample size. Results were reported as odds ratios (OR) with the corresponding 95% confidence interval (95% CI). Metric data were reported as mean value, median, and standard deviation (SD). Differences in categorical and continuous variables were evaluated by a χ^2^ test and Wilcoxon’s signed-rank test, respectively. The level of significance was set at 0.05 (two sided) for all statistical tests.

## Results

In the period between 2015 and 2018, 34 patients (31 men—84% and 3 women—16%) suffering 37 calcaneal fractures were operatively treated. Mean patients’ age was 43.3 years (SD 13.9). Altogether, fourteen of them had prolonged wound healing. *Twenty-two operations were performed with singular application of a pneumatic tourniquet. The mean tourniquet time was 93 min (63–146 min). In both groups there was no quantifiable blood loss.*

Baseline characteristics are summarized in Table 2.

**Table 2:** Baseline characteristics of the study population

	No tourniquet	Pneumatic tourniquet	*p*-value	Total
*n*	15	22		37
Age (years), mean/median (SD)	43.1/47.0 (15.0)	43.4/42.0 (13.4)	0.975	43.3/44.0 (13.9)
Male–female ratio	13:2	18:4	0.532	31:6
BMI, mean/median (SD)	24.7/26.0 (3.1)	26.5/25.8 (4.8)	0.337	25.7/26.0 (4.2)
Time to surgery (days), mean/median (SD)	16.7/16.0 (7.9)	11.2/11.5 (4.4)	**0.011**	13.4/13.0 (6.6)
Active smokers (*n*)	9	5	**0.026**	14
Sanders classification (*n*)			0.741	
I	2	1		3
II	7	10		17
III	5	10		15
IV	1	1		2
ASA (*n*)			0.902	
I	5	7		12
II	8	13		21
III	2	2		4
Drug abuse (*n*)	4	2	0.154	6
Prolonged wound healing (*n*)	4	10	0.247	14
Wound revisions (*n*)	1	3	0.503	4
External fixation (*n*)	1	3	0.503	4
Bone substitutes (*n*)	4	5	0.541	9

Bold values indicates significance *p*-value < 0.05

### Blood flow (BF)

**Fig. 1:**
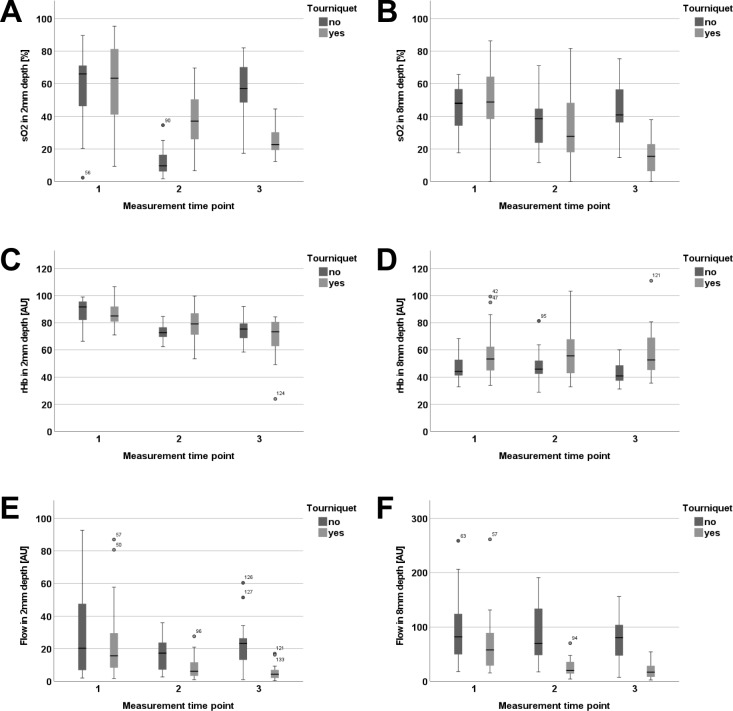
Illustration of changes within the observation period: Oxygen saturation in 2 mm (**A**) and 8mm (**B**) depth. Relative haemoglobin (rHb) concentrations in 2 mm (**C**) and 8mm (**D**) depth. Relative blood flow in 2 mm (**E**) and 8 mm depth (**F**).

Pneumatic tourniquet application resulted in a *significant* decrease in blood flow at 8 mm depth until the end of surgery before tourniquet deflation compared to the control group (flow 8 mm: tourniquet * MTP, p = 0.011; Fig. 1F). In absolute values, blood flow in the tourniquet group decreased from 68.9 AU ± 56.8 (MTP 1) to 18.7 AU ± 13.4 (MTP 3) in the end of the surgery. In contrast, flow values at 2 mm depth did not change over time using the pneumatic tourniquet (flow 2 mm: tourniquet * MTP, p > 0.05, Fig. 1E).

### Tissue oxygen saturation (SO2)

Comparable results were observed with regard to tissue oxygen saturation. The oxygen saturation remained stable at 2 mm depth with use of a pneumatic tourniquet (SO_2_ 2 mm: tourniquet * MTP, p > 0.05) (Fig. 1A), whereas saturation in 8 mm depth decreased significantly (SO_2_ 8 mm: tourniquet * MTP, p < 0.023) compared to the control group (Fig. 1B). In absolute values, SO_2_ at 8 mm depth decreased until the end of the surgery from 48.6% ± 21.5 to 16.6% ± 12.3 (Table 3).

**Table 3:** Predictive values at MTP 3 compared to baseline in logistic regression analyses

Variable	OR	95% CI
SO_2_ 2 mm	0.98	0.944–1.015
SO_2_ 8 mm	0.97	0.928–1.004
F 2 mm	1.00	0.947–1.044
BF 8 mm	0.99	0.977–1.011
rHb 2 mm	1.03	0.972–1.095
rHb 8 mm	1.05	0.997–1.110

### Tissue haemoglobin content (rHb)

The application of a tourniquet did not influence rHb levels over the time course (rHb 2 and 8 mm: tourniquet * MTP, p > 0.05) (Fig. 1C, D).

### Univariable logistic regression analyses

Given that the groups demonstrated largest differences at MTP 3—compared to baseline—predictive values of these differences were examined by logistic regression analyses. None of the variables revealed a statistically significant effect on the development of a prolonged wound healing.

## Discussion

Primary aim of the current study was to analyse the influence of intraoperative pneumatic tourniquet usage on microcirculation in the skin and subcutaneous tissue during surgical treatment of calcaneal fractures. Surgeries were conducted via an extended lateral approach. The secondary aim of the study was to analyse whether changes in microcirculation affected the time of wound secretion/healing after ORIF of calcaneal fractures.

The main results demonstrated that:

- Inflation of a pneumatic tourniquet leads to a deoxygenation and decrease of the blood flow around the extended lateral approach in 8mm depth, whereas no significant changes were observed in the superficial tissues (2 mm depth) or in the decomposition of haemoglobin measured by rHb.
- Intraoperatively decreased microcirculation in deep tissue layers using a tourniquet system did not influence postoperative wound healing secretion time.

Pneumatic tourniquet systems are widely used during orthopaedic trauma surgeries in the extremities. In the context of surgical treatment of intra-articular calcaneal fractures via an extended lateral approach, the application of a tourniquet facilitates the preparation and fracture reduction by rendering a bloodless operation field. However, despite its wide use, relevant complications are attributed to the application of tourniquets, such as pathogenesis, prolonged tissue desaturation, and consecutive enhancement of the hypoxia transcriptome and local acidosis, all of them being under discussion.

With regard to foot and ankle surgery, the effect of the application of a tourniquet on tissue oxygenation was investigated by Shadgan et al. among others [24]. The authors evaluated the tissue oxygenation distal to the tourniquet and compared the results with measurements from the contralateral leg [24], while Lin et al. measured over time the oxygen saturation proximally and distally of the tourniquet as well as on the contralateral leg [25]. Their results are in line with the results for the deeper layer in our study, even if the measurements were not performed in immediate proximity to the surgical approach. They showed a rapid desaturation after *inflation* of the tourniquet followed by a slower continuous desaturation over the time and a rapid saturation, e.g. a hypersaturation after deflation. We also measured a decreased blood and tissue oxygenation at 8mm depth around the extended lateral approach after tourniquet inflation. This is in line with known responses of blood vessels responsible for microcirculation to a hypoxic microenvironment. Various signalling pathways that ultimately lead to relaxation of vascular smooth muscle cells and subsequent vasodilation have been described [26, 27].

Moreover, the blood supply of the lateral hindfoot is subjected to special conditions. On the one hand, the vascular supply of the flap created by the extended lateral approach is mainly dependent on the lateral calcaneal branch of the peroneal artery (LCBP). The macrocirculation delivered by this branch seems to play a major role for wound healing: Bibbo et al. treated 90 calcaneal fractures with an extended lateral approach and reported relevant wound complications in only 6% of cases [28]. All these patients had an absent LCBP Doppler signal prior to surgery. However, despite the larger vessels, specific microcirculatory changes can be detected after calcaneal fractures too. Our work group already reported that the oxygen saturation and blood flow in the tissue along the hypothetically extended lateral approach were lower in patients with calcaneal fractures versus healthy volunteers [16, 29].

Many circumstances accompanying microcirculatory disturbance (e.g. smoking, advanced age and diabetes) were reported as risk factors for wound complications after surgical treatment of calcaneal fractures [13]. Smoking caused significant increases in SO_2_, Hb, and blood flow in the superficial layer of the skin, while diabetes decreased blood flow and increases SO_2_ skin concentrations after surgery [30]. *In addition, norepinephrine delivered as a medication to increase the blood pressure of patients in a more severe state has been shown to decrease the SO2 in the fracture gap of tibial fractures *[31]

In a *previous* study, we identified the level of preoperative oxygen saturation as a good predictor of wound healing complications [16]. This implicates that an additional interference of the intra- and postoperative microcirculation might be a relevant factor for wound healing problems. In this regard, the tourniquet was identified to be a predictive factor for wound hypoxia in total knee replacement accompanied by an increased rate of wound healing complications after tourniquet use [32]. Our study is the first one analysing the influence of the tourniquet-related microcirculatory changes on wound healing in calcaneal fracture treatment by an extended lateral approach. We could not detect any influence of either the reduced deep layer intraoperative saturation or the flow on the occurrence of wound healing problems.

Different mechanisms may be accountable for the missing influence. The median duration of inflated tourniquet was 94 (*range* 63–145) minutes. This might have been too short to induce significant ischaemic tissue impairment. It is known that most of the tourniquet-related complications are time dependent, but the time limit is still controversial. Ninety minutes were described as harmless interval in many publications, but this is only based on animal studies [25]. Gidlof et al. demonstrated that tourniquet-induced ischaemia of 90–180 min led to a progressively worsening endothelial injury [33]. It is obvious that extreme ischaemia of more than 4 h duration can lead to irreversible skeletal muscle injury [33]. However, in addition to the ischemia’s duration, the damage is also determined by the metabolic demand of the tissue. In free flap surgery, hyperaemia and simultaneous reductions in tissue oxygen saturation were observed due to an increased oxygen demand during the first 72 h after anastomosis [34]. The tissues of the lateral hind foot in calcaneal fracture patients might be adapted to lower oxygen saturation due to the disturbance of microcirculation by the fracture itself [16, 29]. The latency between trauma and operation was on average 13 ± 6 (2–41) days [16], which might be enough time to induce intracellular metabolic activity towards an enhanced anaerobic metabolism. This performance adaptation to hypoxic conditions is similar to adaptations observed in high-altitude conditions, being mainly attributable to changes in mitochondrial physiology and leading to greater yield, respiratory capacity for oxidative phosphorylation, and O_2_ affinity [35–39]. Another factor inherent to specific tissues is the ‘microvascular intrinsic heterogeneity’, leading to local oxygen supply shortages due to the fact that the maximum diffusion distance for oxygen delivery from blood vessels into oxygen-consuming tissue is only 20–100 μm [36, 40]. In case of tissue swelling and haematoma formation, the distances between capillaries may grow to an extent exceeding the normal diffusion distance. Of note, the tissues of the heel are capable of sustaining hypoperfusion due to pressure when standing during extended time periods and are known to introduce/provoke reactive hyperaemia when the pressure is released [41]. These seem to be the main reasons for the finding in the present study that the intraoperative decrease of microcirculation had no influence on the postoperative wound healing.

## Limitations

This study has several limitations.

The small number of included patients in this study may be the cause for lack of statistical power and thereby the influence of the tourniquet on wound healing might be underestimated. *Furthermore, the number of smokers was higher in the non-tourniquet group as well as the latency before surgery which might also have had an impact on the rate of wound healing problems. Therefore, multicentric studies with larger sample sizes are necessary to prove our results. With an extended follow up bone healing could be assessed as another endpoint.*

Wound healing might be relevantly influenced by factors which were not analysed in our study. *It has to be kept in mind that especially factors like initial soft tissue damage, fracture type and postoperative infection are able to influence the course of wound healing.* The above-mentioned potential influence of the lateral calcaneal branch of the peroneal artery (LCBP) is another example. The presence of this artery was not proved in our study. *Patients with* chronic limb-threatening ischaemia *(CLTI) were excluded anamnestically but did not perform any specific test.*

In addition, the rate of wound healing problems in the analysed study population was rather high compared to the majority of the literature. However, the complication rates across the literature differ widely. Complication rates from 1.7 to 26.7% were reported. Especially different pre-, intra-, and post-operative management concepts as well as different study populations may be responsible for these differences but also different definitions of complications and indications for revision surgery. Regarding our definition, each prolonged wound secretion later than the seventh postoperative day or *presence of* a simple wound edge necrosis was considered a wound-healing complication.

## Conclusion

Tourniquet application leads to a decrease of blood flow and oxygen saturation in the deeper tissue layers around the region of the extended lateral approach in calcaneal fracture surgery. The intraoperative decrease of microcirculation, however, demonstrated no influence on the postoperative wound healing. The uncritical values for duration and pressure of tourniquet inflation in calcaneal ORIF need to be determined in future studies.

## Data Availability

The data that support the findings of this study are available from the corresponding author, [MK], upon reasonable request.

## Abbreviations

BF [AU]: Blood flow
SO_2_[%]: Tissue oxygen saturation
rHb[AU]: Relative amount of haemoglobin
LMM: Linear mixed model
ORIF: Open reduction and internal fixation
MTP: Measurement time points
LCBP: Lateral calcaneal branch of the peroneal artery
